# Tumor Necrosis Factor Receptor-2 Signals Clear-Cell Renal Carcinoma Proliferation via Phosphorylated 4E Binding Protein-1 and Mitochondrial Gene Translation

**DOI:** 10.1016/j.ajpath.2024.02.019

**Published:** 2024-03-25

**Authors:** Rafia S. Al-Lamki, Aviva M. Tolkovsky, Mohammad Alawwami, WanHua Lu, Sarah F. Field, Jun Wang, Jordan S. Pober, John R. Bradley

**Affiliations:** ∗ Department of Medicine, National Institute of Health Research Cambridge Biomedical Research Centre, University of Cambridge, Cambridge, United Kingdom; † Department of Clinical Neurosciences, The Clifford Allbutt Building, University of Cambridge, Cambridge, United Kingdom; ‡ Dementia Research Institute, Island Research Building, University of Cambridge, Cambridge, United Kingdom; § Department of Immunobiology, Yale University, New Haven, Connecticut

## Abstract

Clear-cell renal cell carcinoma (ccRCC), a tubular epithelial malignancy, secretes tumor necrosis factor (TNF), which signals ccRCC cells in an autocrine manner via two cell surface receptors, TNFR1 and TNFR2, to activate shared and distinct signaling pathways. Selective ligation of TNFR2 drives cell cycle entry of malignant cells via a signaling pathway involving epithelial tyrosine kinase, vascular endothelial cell growth factor receptor type 2, phosphatidylinositol-3-kinase, Akt, ^pSer727^-Stat3, and mammalian target of rapamycin. In this study, phosphorylated 4E binding protein-1 (4EBP1) serine 65 (^pSer65^-4EBP1) was identified as a downstream target of this TNFR2 signaling pathway. ^pSer65^-4EBP1 expression was significantly elevated relative to total 4EBP1 in ccRCC tissue compared with that in normal kidneys, with signal intensity increasing with malignant grade. Selective ligation of TNFR2 with the TNFR2-specific mutein increased ^pSer65-^4EBP1 expression in organ cultures that co-localized with internalized TNFR2 in mitochondria and increased expression of mitochondrially encoded COX (cytochrome *c* oxidase subunit) Cox1, as well as nuclear-encoded Cox4/5b subunits. Pharmacologic inhibition of mammalian target of rapamycin reduced both TNFR2-specific mutein–mediated phosphorylation of 4EBP1 and cell cycle activation in tumor cells while increasing cell death. These results signify the importance of ^pSer65^-4EBP1 in mediating TNFR2-driven cell-cycle entry in tumor cells in ccRCC and implicate a novel relationship between the TNFR2/^pSer65^-4EBP1/COX axis and mitochondrial function.

Renal cell carcinoma (RCC) is the most common type of kidney cancer in adults, accounting for approximately 90% of all adult renal malignancies and 2% to 3% of all cancers.^1^, ^2^, ^3^ The clear-cell RCC (ccRCC) represents up to 75% of all RCC cases^4^ and originates from tubular epithelial cells. ccRCC tumor cells secrete tumor necrosis factor (TNF), which acts as an autocrine growth factor.^5^ TNF signals via two cell surface receptors, TNFR1 and TNFR2, which mediate both shared and distinct signaling pathways. Selective activation of TNFR2 drives ccRCC tumor cell proliferation by a signaling pathway involving cytosolic endothelial/epithelial tyrosine kinase–mediated activation of vascular endothelial cell growth factor receptor type 2, phosphatidylinositol-3-kinase (PI3K), Akt, and the mammalian target of rapamycin (mTOR).^5^ Subsequent studies show that TNFR2 also promotes the survival of RCC specifically through serine 727 phosphorylation of Stat 3 (pStat3^S727^). Devoid of tyrosine phosphorylation, it translocates to mitochondria rather than the cell nucleus, where it co-localizes with internalized TNFR2.^5^, ^6^, ^7^ Inhibition of any of the involved kinases or siRNA knockdown of TNFR2 or STAT3 promotes cell death associated with mitochondrial morphologic changes, cytochrome *c* release, and generation of reactive oxygen species.^7^

The present study further explored the role of mTOR in the TNF protumor response. mTOR is a major driver of many cancers, including RCC,^8^^,^^9^ and is an emerging therapeutic target.^10^, ^11^, ^12^, ^13^ mTOR is a conserved serine/threonine protein kinase that exists in two different complexes, mTOR complex 1 (mTORC1) and mTORC2.^14^ mTORC1 promotes protein synthesis, partly through cap-dependent translation mediated by 4E binding protein-1 (4EBP1),^15^^,^^16^ whose mTORC1-dependent phosphorylation enhances mitochondrial function by increasing translation of nuclear-encoded mitochondrial-related mRNAs.^17^ Aberrant activation of the mTOR protein translational pathway presents as either a gain of growth-promoting function or a loss of inhibitory function and may affect the cell dynamics facilitating neoplastic transformation.

The present study further investigated TNFR2-mediated mTOR-dependent signaling pathways that impinge on protein synthesis by focusing on 4EBP1, prompted by the identification of 4EBP1 phosphorylated at serine-65 (^pSer65-^4EBP1) as a responder to selective TNFR2 activation using a phosphorylated proteome array. In ccRCC tissue, significantly higher expression of ^pSer65-^4EBP1 occurred in tumor cells than in nontumor tissue (NK) cells, with the extent of phosphorylation increasing with malignant grade. In organ cultures of ccRCC, TNFR2 stimulation increased ^pSer65-^4EBP that co-localized with TNFR2 in tumor cells, which was primarily confined to the cytoplasm and mitochondria. Moreover, 4EPB1 phosphorylation was correlated with increased expression of the cytochrome *c* oxidase (COX) subunits Cox1, Cox4, and Cox5b. Pretreatment of organ cultures with mTORC1/2 inhibitors Torin 2 or Ku63794 before TNFR2 stimulation led to a significant reduction of ^pSer65-^4EBP1 expression that correlated with cell cycle inhibition. Thus, ^pSer65^-4EBP1 is likely to be an important mediator of TNFR2-driven cell-cycle activation in ccRCC tumor cells, possibly by providing an increased mitochondrial energy reserve.

## Materials and Methods

### General Reagents

Primary antibodies used in this study are listed in Table 1. Species-specific fluorescent secondary antibodies were conjugated with either NorthernLights (R&D Systems, Abingdon, UK) or AlexaFluor (Invitrogen, Paisley, UK). Goat anti-rabbit horseradish peroxidase–conjugated antibody (catalog number P0448; Dakocytomation, Ely, UK); Vectashield anti-fade mounting media (catalog number H-1000; 2BScientific, Oxfordshire, UK); Clarity Western ECL Substrate (catalog number 170-5060; BioRad Laboratories Ltd, Hertfordshire, UK) or SuperSignal West Dura ECL substrate (catalog number 34075; Thermo Fisher Scientific, Paisley, UK); terminal transferase enzyme (catalog number 03333566001; Roche Diagnostics, Mannheim, Germany); and Hoechst-33342 (catalog number 62249; Thermo Fisher Scientific) were obtained. TNFR2-specific mutein (R2TNF) containing the point mutation D143N^18^ was a generous gift from Professor Peter Vandenabeele (VIB, Ghent, Belgium). R2TNF is a recombinant mutation of the TNF sequence that enables the mutated protein to bind selectively to TNFR2. Inhibitors were dissolved in dimethyl sulfoxide and stored at −20°C or −80°C.

**Table 1:** List of Primary Antibodies Used in This Study

Antibody	Catalog no.	Supplier	Application
Rabbit anti-TNFR2	28746-1-AP	Proteintech (Manchester, UK)	IF
Mouse anti-HSP60	66041-1-1g	Proteintech	IF
Mouse anti-total 4EBP1	60246-1-1g	Proteintech	IF, IB
Mouse anti-Tom20	11802-1-AP	Proteintech	IF
Mouse anti-Cox1	A6403	Thermo Fisher Scientific	IF
Goat anti-TNFR2	AB-226-PB	R&D Systems	IF
Human β-actin	MAB8969	R&D Systems	IB
Mouse anti–RCC-MA	NCL-RCC 2011-03	Novocastra (Newcastle Upon Tyne, UK)	IF
Rabbit anti–phosphorylated 4EBP1 S65	CSB-PA007991	Cusabio Technology (Houston, TX)	IF
Rabbit anti–phosphorylated 4EBP1 S65	A16404	Antibodies (Cambridge, UK)	IF
Rabbit anti–phosphorylated 4EBP1 S65	9451	Cell Signaling (Danvers, MA)	IF, IB
Mouse anti–phosphorylated 4EBP1 T37/46	S2855	Cell Signaling	IF, IB
Rabbit anti–phosphorylated 4EBP1 S65	AP0032	ABclonal (Woburn, MA)	IF
Mouse anti-Cox5b	A21349	Thermo Fisher Scientific	IF
Mouse anti-Cox4	A21348	Thermo Fisher Scientific	IF
Mouse anti–phosphorylated histone H3^S10^	ab14955	Abcam (Cambridge, UK)	IF
Rabbit anti–phosphorylated histone H3^S10^	ab5176	Abcam	IF

Cox, cytochrome *c* oxidase; HSP, heat shock protein; IB, immunoblotting; IF, immunofluorescence; RCC-MA, a tumor marker; Tom20, translocase of outer mitochondrial membrane; TNFR, tumor necrosis factor receptor.

### Collection of Tissue Samples

Samples of ccRCC and corresponding NK (categorized as histologically normal kidney cortex in sites remote from the tumor) from radical nephrectomies removed for tumor resection were collected through the Cambridge University Hospital Tissue Bank (Cambridge, UK). Written informed consent was obtained from all patients in accordance with the local Ethics Committee. ccRCC samples were scored and graded on hematoxylin and eosin–stained sections as Fuhrman grade 1 (*n* = 4), grade 2 (*n* = 20), grade 3 (*n* = 16), and grade 4 (*n* = 20) by a pathologist according to the World Health Organization/International Society of Urological Pathology.^19^ High-grade tumors with extensive areas of necrosis were excluded from the analysis. All samples were either fixed overnight in 4% formaldehyde in 0.1 mol/L phosphate buffer, pH 7.6, at 4°C and paraffin wax embedded for immunofluorescence or snap frozen in isopentane-cooled liquid nitrogen and stored at −80°C until use. Parallel unfixed fresh tissue was processed for organ culture experiments. Sections (5 μm thick) were prepared for subsequent analysis.

### ccRCC and NK Organ Cultures

Organ culture protocols were performed as previously described.^6^^,^^20^^,^^21^ In brief, duplicate <1-mm^3^ fragments of fresh tissue from ccRCC (grades 1 to 2) and NK (*n* = 5 per study group) were immersed in M199 medium containing 10% heat-inactivated fetal calf serum, antibiotics, and 2.2 mmol/L glutamine. Cultures were either left in media alone [untreated (UT) controls] or treated with R2TNF (1 μg/mL), for 3 hours at 37°C.^5^^,^^6^^,^^18^^,^^21^ Parallel cultures were pretreated with either cycloheximide (20 μg/mL) or the mTOR inhibitors, Torin 2, Ku63794, or rapamycin (50 μmol/L), for 1 hour before R2TNF addition. Samples were fixed and paraffin embedded, and sections (5 μm thick) were prepared for subsequent analysis.

### Immunofluorescence

Sections of cRCC, NK, and corresponding organ cultures were immunostained using previous protocols^5^^,^^6^^,^^21^ following 2 minutes of high-pressure antigen retrieval with acid buffer (0.01 mol/L sodium citrate buffer, pH 6.0). Samples were incubated with primary antibodies (1:100) for 1 hour at room temperature, followed by NorthernLight- or AlexaFluor-conjugated secondary antibodies (1:200) at room temperature containing Hoescht 33342 (1 μg/mL) for nuclei detection. For negative controls, the primary antibody was replaced with an isotype-specific serum. Sections were mounted in Vectashield and viewed in a blinded manner by two observers (R.S.A.-L. and A.M.T.) under a Leica TCS-SPE confocal microscopy system (Leica Microsystems, Milton Keynes, UK) or a Zeiss LSM 880 Airyscan (Carl Zeiss, Cambourne, UK) for super-resolution imaging. Images for each fluorophore were acquired sequentially using the same constant acquisition time and settings and processed using Adobe Photoshop PS version 24.4.1 (Adobe Systems, Maidenhead, UK). As previously described,^22^ the intensity of fluorescence per cell was calculated as the corrected total cell fluorescence [CTCF = integrated density – (area of selected cell × mean fluorescence of background readings)] using ImageJ software version 1.53k (NIH, Bethesda, MD; *http://imagej.nih.gov/ij*). For each set of antibodies, three slides from the same patient sample were processed, and the numbers quantified were combined into a single value. *N* represents the number of independent patient samples out of a pool of four cases for primary tissue and five cases for tissue organ cultures.

### Immunoblot Analysis

ccRCC and NK tissues were lysed in radioimmunoprecipitation assay buffer, cleared by centrifugation at 18,000 × *g* for 20 minutes at 4°C, and 30 to 50 μg per sample were separated by SDS-PAGE. Proteins were transferred to a nitrocellulose membrane, blocked with 4% skim milk for 1 hour at room temperature, and probed overnight at 4°C with ^pSer65^-4EBP1 or ^pThr36/47^-4EBP1 (1:1000) followed by secondary horseradish peroxidase–conjugated antibodies. Protein bands were visualised using a ChemDoc Universal Hood III Imaging System (BioRad Laboratories Ltd). Relative protein levels were quantified by normalizing to β-actin and Ponceau S staining.^23^

### Immunogold Electron Microscopy

As previously described,^24^ Formvar-coated grids (Agar Scientific; Ted Pella Inc., Redding, CA) containing 50-nm sections of UT and R2TNF-treated ccRCC organ cultures were incubated with antibodies to ^pSer65^-4EBP1 or TNFR2 and heat shock protein 60 or ^pSer65^-4EBP1 and TNFR2 (1:5 dilution) overnight at room temperature and further incubated with secondary antibodies conjugated to 5- and 15-nm colloidal gold particles (British Biocell, Portland, ME; 1:100 dilution), then stained with uranyl acetate and lead citrate before viewing in a Hitachi electron microscope (Hitachi High-Tech, Maidenhead, UK) at an accelerating voltage of 80 kV.

### Terminal Deoxynucleotidyl Transferase-Mediated dUTP Nick-End Labeling

Cell death was detected as previously described.^24^ In brief, sections pretreated with protease were labeled with terminal deoxynucleotidyl transferase-mediated dUTP nick-end labeling (TUNEL) label mix and terminal transferase enzyme, according to the manufacturer's instructions (Roche Diagnostics Corp., Indianapolis, IN). This was followed by staining with Hoechst 33342 for 10 minutes at room temperature and mounting in Vectashield before viewing on the Leica TCS-SPE confocal microscopy system.

### Analysis of Cell Death and Proliferation

The average number of TUNEL-positive tumor cells in ccRCC was counted in 10 random fields of view at ×40 magnification from each treatment divided by the total cell numbers to generate the percentage positive cells. Similarly, the number of ^pSer65^-4EBP1−positive and pH3^S10^-positive tumor cells were counted and divided by the total cell numbers to generate the percentage of positive cells, calculated as the proliferative index for each treatment.

### Statistical Analysis

All data are expressed as means ± SD. All statistical tests were performed using Microsoft (Redmond, WA) Excel for Mac version 16.81 and GraphPad (La Jolla, CA) Prism version 10.1.0. Statistical significance was calculated by an analysis of variance, followed by *post hoc* multiple comparison test assuming a *P* < 0.05 as statistically significant.

## Results

### ^pSer65^-4EBP1 Is Induced in ccRCC Tumor-Derived Tissues and Co-Localizes With TNFR2

**Figure 1:**
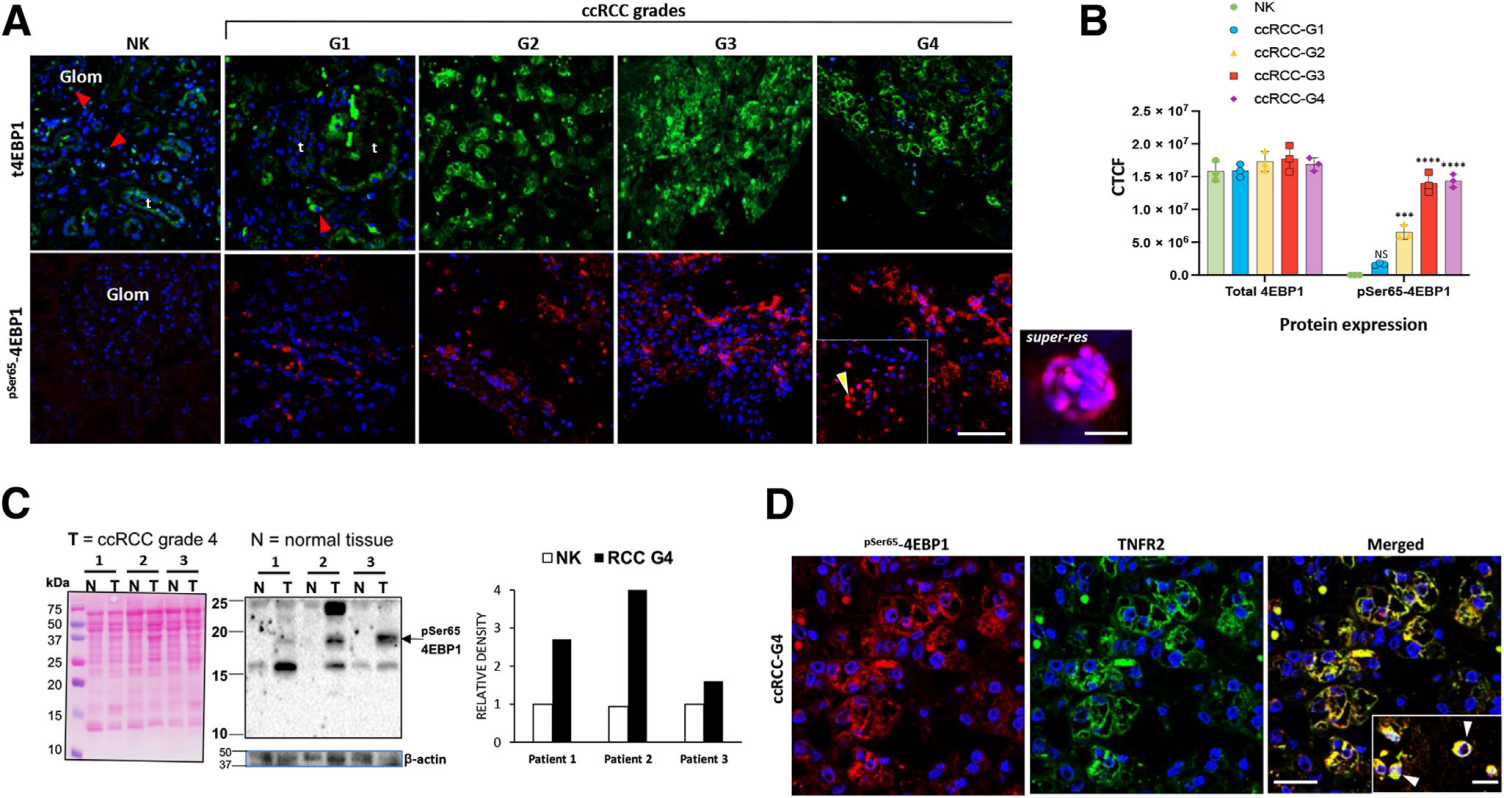
^pSer65^-4EBP1 is abundantly expressed in ccRCC tumor and co-localizes with tumor necrosis factor receptor (TNFR) 2. Representative confocal images of single-immunofluorescence staining of ccRCC tumor grades (G1 to G4) and corresponding nontumor tissue (NK). **A: Top panels:** Staining for total 4EBP1 (t4EBP1). A marked signal was detected in NK, confined to normal tubular cells (t), some isolated cells within glomeruli (Glom), and the interstitium (**red arrowheads**). A similar pattern and intensity were found in tumor cells in all ccRCC grades. **Bottom panels:** Staining for ^pSer65^-4EBP1. Signal was undetectable in NK and infrequently present in G1 ccRCC tumor cells but accumulated with increasing intensity in ccRCC according to malignant grade (G2 to G4). Signal was mainly cytoplasmic but also found infrequently in the nucleus of some tumor cells. **Inset:** low-power image; **arrowhead** indicates the panel labeled *super-res* shows a high-power image of 4E-BP1 staining in the nuclei taken by super-resolution microscopy. **B:** Signal intensity per cell was quantified as corrected total cell fluorescence (CTCF). Two-way analysis of variance was performed. **C:** Immunoblot of lysates from three independent ccRCC G4 tumor resections (T) alongside corresponding nontumor kidney (N) from the same tissue, showing an increased abundance of ^pSer65^-4EBP1 in all three tumors compared with normal tissue. β-Actin and Ponceau S stain show protein loading. Bar graph shows the relative intensity of ^pSer65^-4EBP1 band normalized to N for each pair. **D:** Representative confocal images of ccRCC G4 staining showing co-localization of signal for ^pSer65^-4EBP1 and TNFR2 in tumor cells and in infiltrating cells within the interstitium (**inset**; **arrowheads**). Data are expressed as means ± SD (**B**). *n* = 3 per group with similar results (**A**, **B**, and **D**). ∗∗∗*P* < 0.001 versus NK and G1; ∗∗∗∗*P* < 0.0001 versus NK, G1, and G2. Scale bars: 50 μm (**A** and **D**, **main images**); 25 μm (**D**, **inset**). Original magnification: ×40 (**A**); ×63 (**A**, **super-res image**, and **D, main images and inset**). NS, not significant.

**Supplemental Figure S1:**
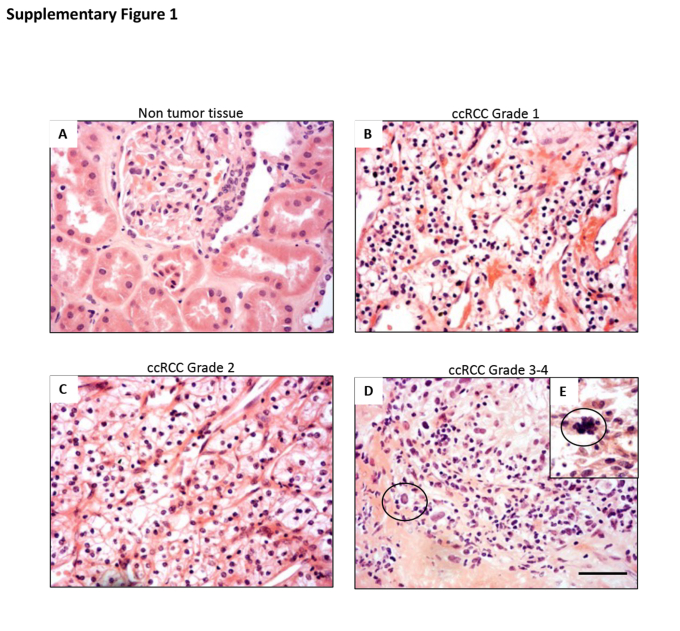
Representative hematoxylin and eosin–stained sections of the nontumor kidney (**A**) and different grades of ccRCC: Fuhrman grade 1 shows a uniform nuclear pattern of tumor cells containing condensed chromatin (**B**); Fuhrman grade 2 shows tumor cells containing prominent medium-sized nuclei with slightly irregular contours with small distinct nucleoli (**C**); and Fuhrman grades 3 and 4 show tumor cells with irregular large to multilobed nuclei and prominent nucleoli (circled; **D** and **E**). Scale bar = 100 μm.

**Supplemental Figure S2:**
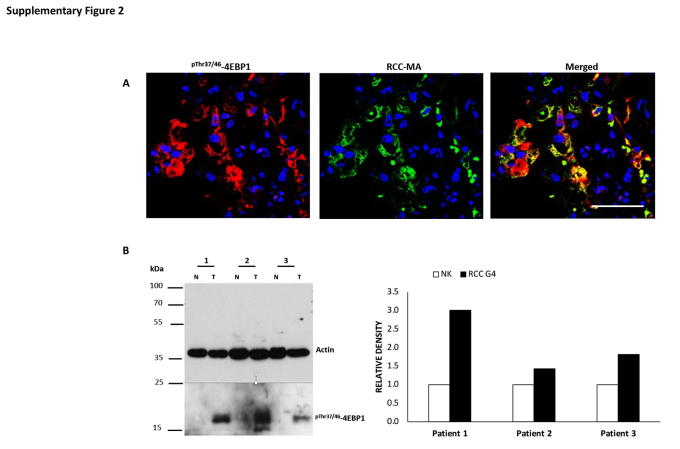
**A:** Representative confocal image of a high-grade tumor [ccRCC grade 4 (G4)] shows a signal for ^pThr37/46^-4EBP1 in tumor cells indicated by RCC-MA (marker of RCC) corroborated by immunoblotting of tissue lysates from three resections, showing the presence of ^pThr37/46^-4EBP1 in the tumor (T) but not in the corresponding nontumor kidney (N and NK). **B: Left panel:** Actin blot shows protein loading. **Right panel:** The intensity of ^pThr37/46^-4EBP1 band in T normalized to that of N for each sample. *n* = 3 per group with similar results (**B**). Scale bar = 50 μm (**A**). Original magnification, ×40 (**A**).

To investigate whether TNFR2-dependent mTOR signaling responses depend on 4EBP1 expression and phosphorylation, patient-derived ccRCC tissue and corresponding NK from the same biopsies were analysed.^6^^,^^7^ Although both regions showed a similar fluorescence signal intensity for total 4EBP1 protein, NK displayed a minimal signal for ^pSer65^-4EBP1 (<1% of cells), but sections of ccRCC showed a significantly higher signal in tumor cells (approximately threefold increase in grade 2 tumors), which further increased as tumor grade progressed (approximately eightfold in grade 4 tumors). Some signal was also detected in nuclei of tumor cells (approximately 15% of positive cells, imaged using high-resolution microscopy) (Figure 1A), with fluorescence intensity quantified as CTCF (Figure 1B). Representative hematoxylin and eosin–stained sections of NK and grade 1 to 4 tumors are presented in Supplemental Figure S1. Immunoblotting of grade 4 tumor extracts also demonstrated an increase in ^pSer65^-4EBP1 expression above that of NK tissue (Figure 1C). 4EBP1 phosphorylation on several sites is necessary for its dissociation from eukaryotic translational initiation 4E (EIF4E), thereby enabling EIF4E to initiate protein translation. As phosphorylation of 4EBP1 at Ser65 is dependent on prior phosphorylation of Thr37/46,^25^^,^^26^ grade 4 tumors were examined for expression of phosphorylated ^Thr37/46^-4EBP1. A significant positive fluorescent signal for phosphorylated ^pThr37/46^-4EBP1 was also found in tumor cells (positive for RCC-MA, a tumor marker) and corroborated by immunoblotting (Supplemental Figure S2). This indicated a state of 4EBP1 phosphorylation capable of initiating protein synthesis downstream of TNFR2 activation. Increased TNFR2 expression in tumor cells in ccRCC has been previously reported.^5^ To investigate whether ^pSer65^-4EBP1 might be associated with elevated TNFR2, grade 4 ccRCC sections were costained for TNFR2 and ^pSer65^-4EBP1. Cytoplasmic costaining was observed in approximately 80% of tumor cells as well as in vascular endothelial cells and in isolated interstitial cells (Figure 1D). Hence, increased ^pSer65^-4EBP1 is associated with increased TNFR2 expression in ccRCC.

### TNFR2 Stimulation Induces Up-Regulation and Co-Localization of TNFR2 and ^pSer65^-4EBP1 in ccRCC Organ Cultures

**Figure 2:**
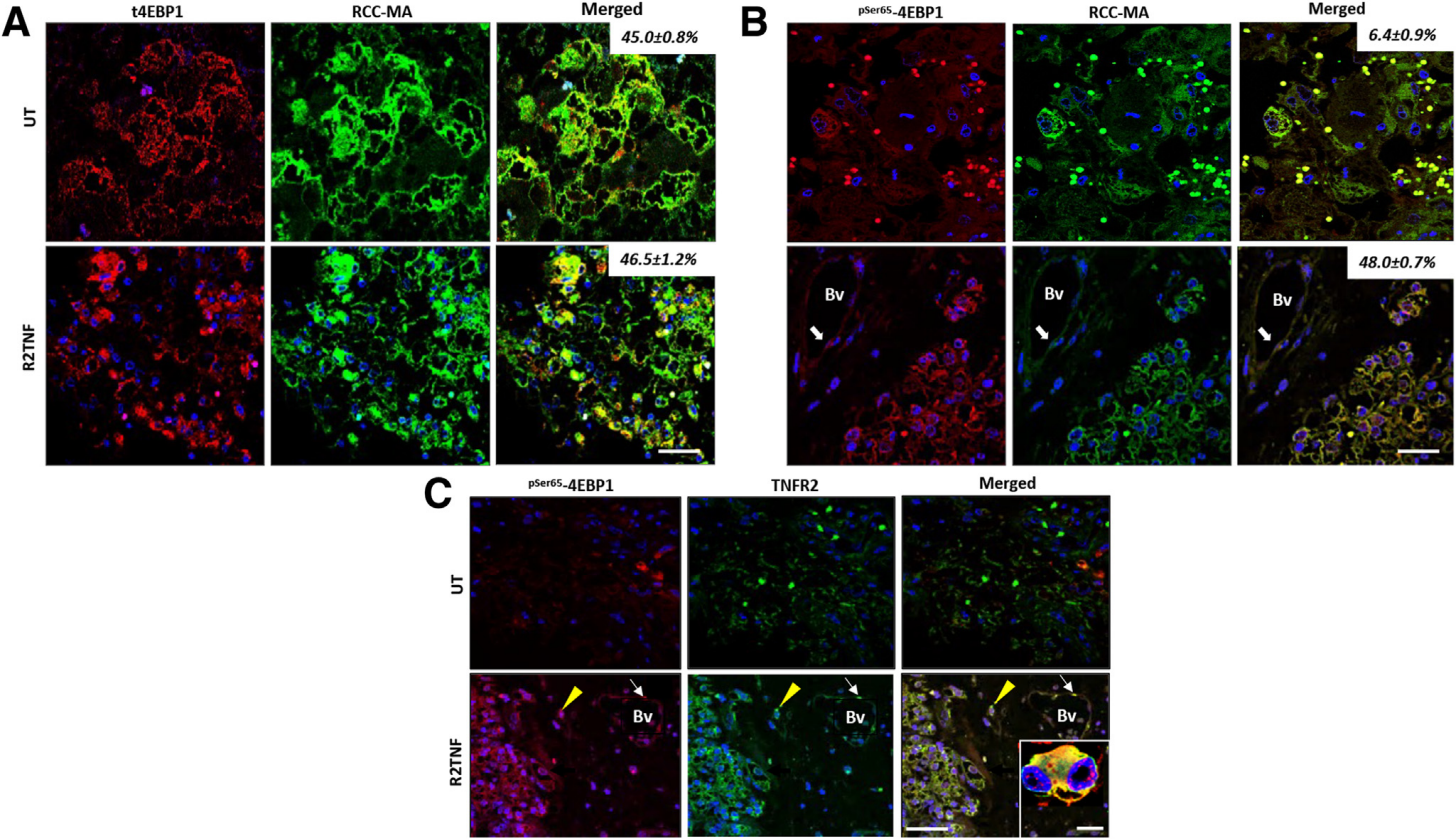
Activation of tumor necrosis factor receptor (TNFR) 2 by TNFR2-specific mutein (R2TNF) induces expression of ^pSer65^-4EBP1 and its co-localization with TNFR2 in tumor cells in organ cultures of ccRCC. **A:** Representative confocal images of combined immunofluorescence in low-grade ccRCC organ culture sections stained for total (t) 4EBP1. A similar signal intensity was found in tumor cells (identified using the tumor marker RCC-MA) in control [untreated (UT)] and R2TNF-treated cultures. **B:** In comparison, R2TNF induced expression of ^pSer65^-4EBP1 in tumor cells with a weak infrequent signal in controls. A signal for ^pSer65^-4EBP1 is also seen in endothelial cells in blood vessels (Bv; **white arrows**), negative for RCC-MA. Numbers indicate percentage co-localization in tumor cells. **C:**^pSer65^-4EBP1 and TNFR2 co-localization in tumor cells, in vascular endothelial cells (Bv; **white arrows**), and in infiltrating cells (**yellow arrowheads**). **Inset**: Cosignal in a bilobed tumor cell in another area on the same section. Data are given as means ± SD (**A** and **B**). *n* = 3 per group with similar results (**A** and **B**). Scale bars: 50 μm (**A**–**C, main images**); 10 μm (**C**, **inset**). Original magnification: ×40 (**A**–**C**, **main images**); ×63 (**C**, **inset**).

To establish whether stimulation of TNFR2 is sufficient to drive phosphorylation of 4EBP1, organ cultures from low-grade tumor tissue were used. Low grade was chosen to avoid possible stimulation of TNFR2 by endogenous sources of TNF.^20^ TNFR2 was activated selectively using an engineered TNFR2 mutein (labeled R2TNF).^18^ Total 4EBP1 was expressed in ccRCC tumor cells (positive for tumor marker RCC-MA^27^) irrespective of TNFR2 stimulation by R2TNF (percentage cell expression: UT, 45.0% ± 0.8%; R2TNF treated, 46.5% ± 1.2%) (Figure 2A). In contrast, R2TNF induced a significant increase in ^pSer65^-4EBP1 expression mainly within the cytoplasm in tumor cells (percentage cell expression: UT, 6.4% ± 0.9%; R2TNF treated, 48.0% ± 0.7%). Similar to advanced-grade ccRCC tissue, some staining was also found in vascular endothelial cells and isolated interstitial cells (Figure 2B and Table 2). The increase in ^pSer65^-4EBP1 expression in response to R2TNF was commensurate with that of TNFR2 in tumor cells, as well as in vascular endothelial cells and isolated interstitial cells (Figure 2C), similar to the increase in both proteins in ccRCC tumor tissue. These data confirm that TNFR2-mediated signals induce phosphorylation of 4EBP1 in ccRCC tumor cells.

**Table 2:** Quantification of the Percentage of t4EBP1 and ^pSer65^-4EBP1 Expression in ccRCCoC and Corresponding NKoC Either Left UT or Treated With R2TNF

Treatment	NKoC			ccRCCoC		
	t4EBP1					
	nTEC	BvEC	ICs	mTEC	BvEC	ICs
UT	42.2 ± 1.0	41.1 ± 0.8	31.4 ± 1.3	45.0 ± 0.8	43.2 ± 0.4	34.1 ± 0.5
R2TNF	42.3 ± 0.6	43.2 ± 0.3	32.1 ± 0.2	46.5 ± 1.2	47.1 ± 0.3	36.1 ± 1.2


Treatment	NKoC			ccRCCoC		
	^pSer65^-4EBP1					
	nTEC	BvEC	ICs	mTEC	BvEC	ICs
UT	3.0 ± 0.5	2.7 ± 0.9	2.4 ± 0.4	6.4 ± 0.9	6.3 ± 0.2	5.9 ± 0.3
R2TNF	15.1 ± 0.4∗	17 ± 0.6∗	15.2 ± 0.3∗	48.0 ± 0.7†	45.0 ± 0.4†	42 ± 0.5†


Values are means ± SD percentages from three independent patient samples. t4EBP1 expression is present in all cultures. In comparison, R2TNF induced a marked ^pSer65-^4EBP1 expression in TECs, BvECs, and ICs, with a less pronounced effect in R2TNF-treated NKoC.

BvEC, vascular endothelial cell; ccRCCoC, organ cultures of ccRCC; IC, isolated interstitial cell; mTEC, malignant tubular epithelial cell; NKoC, organ cultures of nontumor kidney; nTEC, normal tubular epithelial cell; R2TNF, TNFR2 mutein; tEBP1, total EPB1; UT, untreated.

∗ NKoC: *P* < 0.001 versus UT.

† ccRCCoC: *P* < 0.0001 versus UT.

### ccRCC Organ Cultures Treated with TNFR2 Stimuli Show Co-Localization of TNFR2 or ^pSer65^-4EBP1 in Mitochondria and Induction of Nuclear and Mitochondrially Encoded Genes

**Figure 3:**
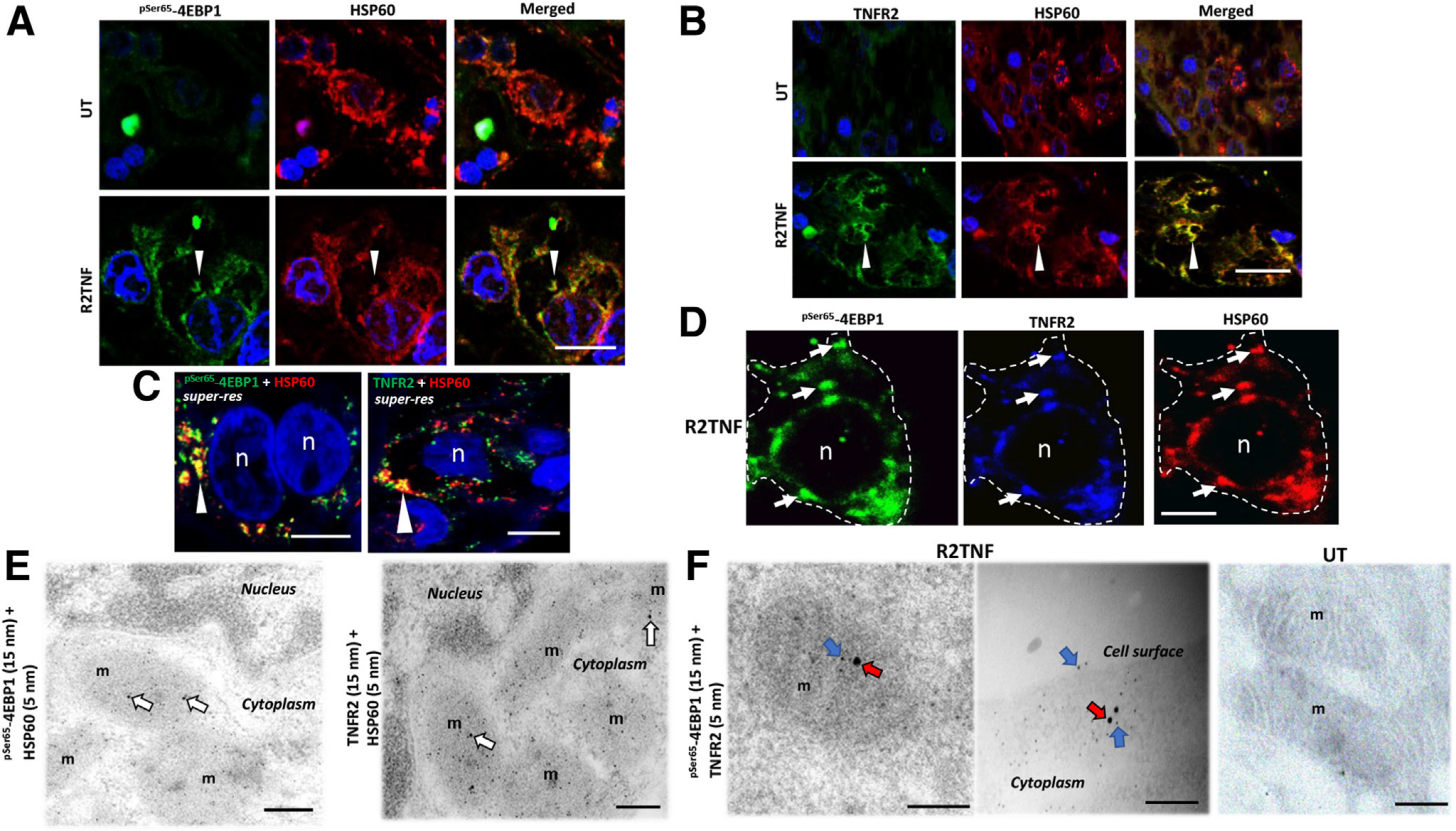
Tumor necrosis factor receptor (TNFR) 2–specific mutein (R2TNF) induces co-localization of ^pSer65^-4EBP1 and TNFR2 in mitochondria (m). Representative panels of high-power confocal images of combined immunofluorescence (IF). **A** and **B:**^pSer65^-4EBP1 and heat shock protein (HSP) 60 (mitochondrial matrix protein; **A**) or TNFR2 and HSP60 (**B**) in low-grade ccRCC organ culture sections. Images show R2TNF-induced co-localization of the two proteins in mitochondria in tumor cells (**arrowheads**). **C** and **D:** Super-resolution images (*super-res)* of tumor cells show mitochondrial co-localization of the two proteins (**white arrowheads**; **C**), confirmed by triple IF (**D**), which shows ^pSer65^-4EBP1/TNFR2/HSP60^+^ (**arrows**) within a tumor cell (**white dotted lines**). **E:** Representative immunogold electron micrographs show presence of ^pSer65^-4EBP1 and TNFR2 (15-nm colloidal gold) in mitochondria, labeled with HSP60 (5-nm colloidal gold) on R2TNF-treated low-grade ccRCC organ sections (**white arrows**). **F:** The close proximity of ^pSer65^-4EBP1 (**red arrows**) and TNFR2 (**blue arrows**) in mitochondria and the presence of TNFR2 on the cell surface compared with untreated (UT) cultures, which show a rare signal. *n* = 3 per group with similar results (**A**–**F**). Scale bars: 25 μm (**A** and **B**); 75 μm (**C** and **D**); 200 nm (**E** and **F, left** and **right panels**); 500 nm (**F**, **middle panel**). Original magnifications: ×40 (**A** and **B**); ×63 (**C** and **D**). N, nucleus.

As TNFR2 has been previously identified in mitochondria, whether TNFR2-mediated expression of ^pSer65^-4EBP1 was also observed together with TNFR2 in mitochondria was investigated next. Co-localization of the two proteins in mitochondria in tumor cells was confirmed by costaining for heat shock protein 60 (a mitochondrial matrix protein) by immunofluorescence (Figure 3, A–D) and immunogold electron microscopy (Figure 3, E and F).

**Figure 4:**
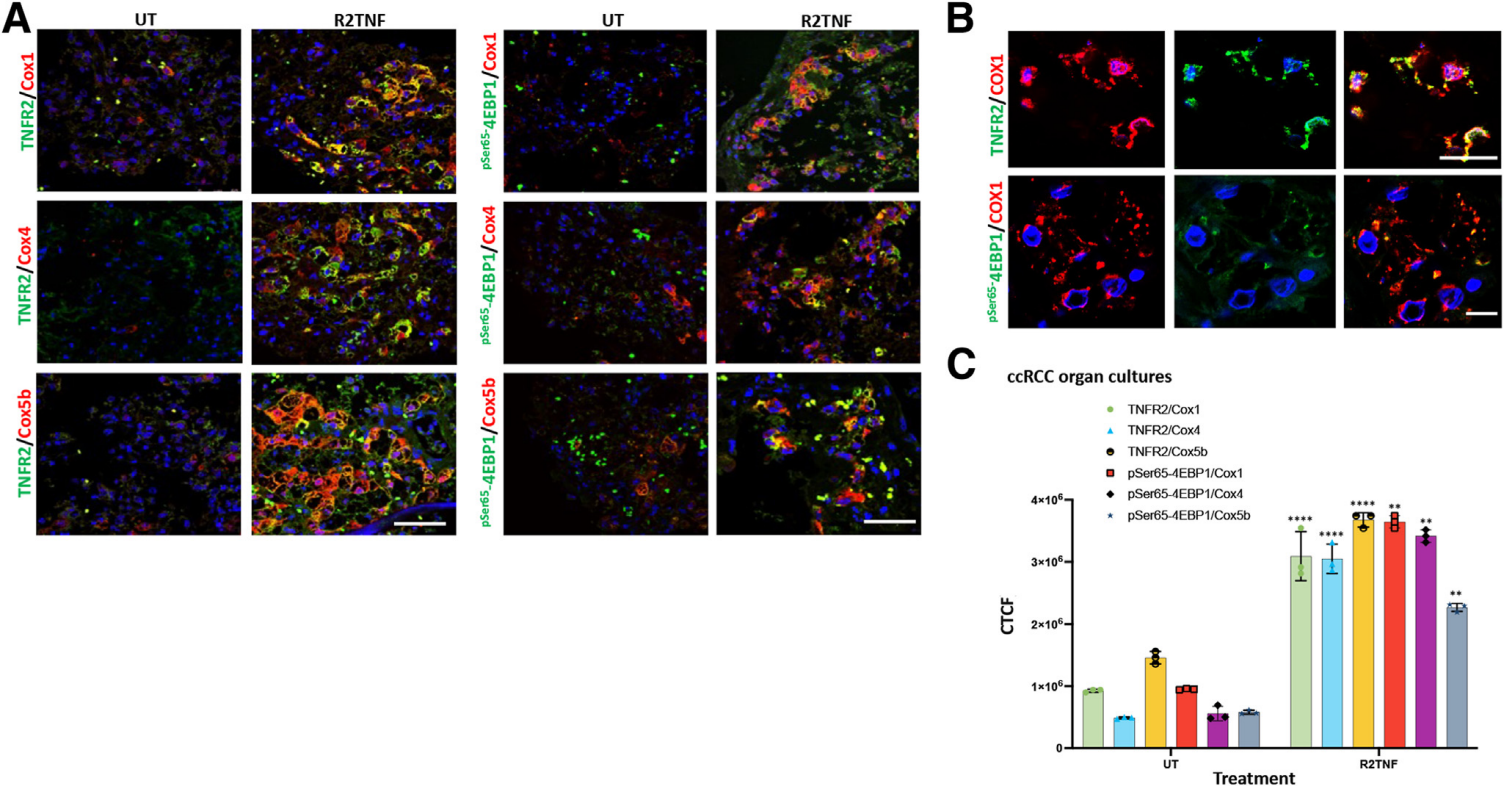
Tumor necrosis factor receptor (TNFR) 2–specific mutein (R2TNF) induces co-localization of ^pSer65^-4EBP1 and TNFR2 with cytochrome *c* oxidase (Cox) subunits. **A:** Representative confocal images of combined immunofluorescence of ^pSer65^-4EBP1 or TNFR2 with Cox1, Cox4, or Cox5b in low-grade ccRCC organ culture sections show R2TNF-induced cosignal of ^Ser65^-4EBP1 or TNFR2 and Cox1, Cox4, and Cox5. **B:** High-power images show R2TNF-induced cosignal of ^Ser65^-4EBP1 or TNFR2 and Cox1. **C:** Quantification of signal intensity of R2TNF-induced cosignal compared with untreated (UT) presented as corrected total cell fluorescence (CTCF). Two-way analysis of variance was performed. Data are given as means ± SD (**C**). *n* = 3 per group with similar results (**C**). ∗∗*P* < 0.01, ∗∗∗∗*P* < 0.0001 versus controls. Scale bars: 50 μm (**A**); 25 μm (**B**, **top panels**); 10 μm (**B**, **bottom panels**). Original magnification: ×40 (**A**); ×63 (**B**).

**Supplemental Figure S3:**
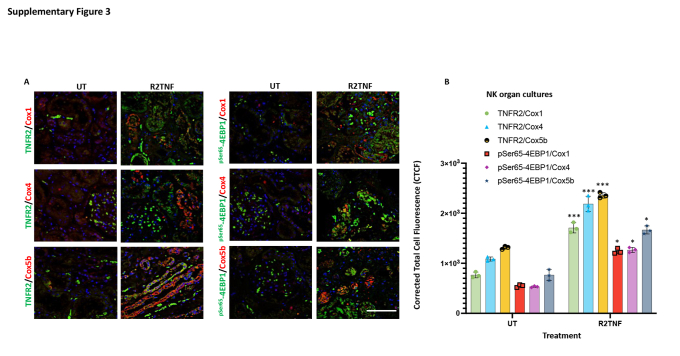
Representative confocal images of combined immunofluorescence for ^pSer65^-4EBP1 or tumor necrosis factor receptor (TNFR) 2 and cytochrome *c* oxidase (Cox) 1, Cox4, or Cox5b on organ culture sections from nontumor kidney either left untreated (UT) or treated with TNFR2-specific mutein (R2TNF; **A**), quantified as corrected total cell fluorescence (**B**). Two-way analysis of variance was performed. Note that although R2TNF addition caused an increase in both ^pSer65^-4EBP1 and COX subunits, the actual intensity relative to that in tumor tissue was 100-fold less. Data are expressed as means ± SD (**B**). *n* = 3 per group with similar results (**B**). ∗*P* < 0.05, ∗∗∗*P* < 0.001 versus UT. Scale bars = 50 μm (**A**). Original magnification, ×40 (**A**).

The mTORC1/4EBP1 pathway augments cellular energy homeostasis via the translation of nucleus-encoded mitochondria-related mRNAs.^17^ To examine whether mitochondrial-related protein translation may also be implicated in the mitochondrial localization of ^pSer65^-4EBP1, the effect of R2TNF-mediated signaling on expression of Cox1, a mitochondrially encoded subunit of cytochrome *c* oxidase (complex IV), as well as the nuclear-encoded subunits Cox4 and Cox5b was examined. In organ cultures of ccRCC, stimulation by R2TNF caused a significant increase in Cox1, Cox4, and Cox5b expression compared with UT cultures (approximately 3.2-fold, approximately 3.7-fold, and approximately 2.7-fold, respectively) that co-localized in cells with elevated TNFR2, and a similarly significant increase in Cox1, Cox4, or Cox5b expression (approximately 3.7-fold, approximately 6.7-fold, and approximately 4.2-fold, respectively) that co-localized in cells with elevated ^pSer65^-4EBP1 (Figure 4, A and B), quantified as CTCF (Figure 4C). R2TNF induced a smaller change (<2.5-fold) in expression and co-localization of the COX subunits in NK. However, this occurred on a signal intensity background that was approximately 1000-fold less intense than that of ccRCC tissue (maximum CTCF intensity in NK, approximately 2.5 × 10^3^ compared with approximately 4 × 10^6^ in ccRCC) (Supplemental Figure S3). Thus, TNFR2-mediated signaling may be involved in the translational regulation of mitochondrial as well as in nuclear-encoded mitochondrial genes in ccRCC, with the former, perhaps, being mediated by mitochondrial-targeted TNFR2/^pSer65^-4EBP1.

### Cycloheximide Inhibits Expression of Nuclear-Encoded Subunits Cox4/5b but Not that of Mitochondrially Encoded Cox1

**Figure 5:**
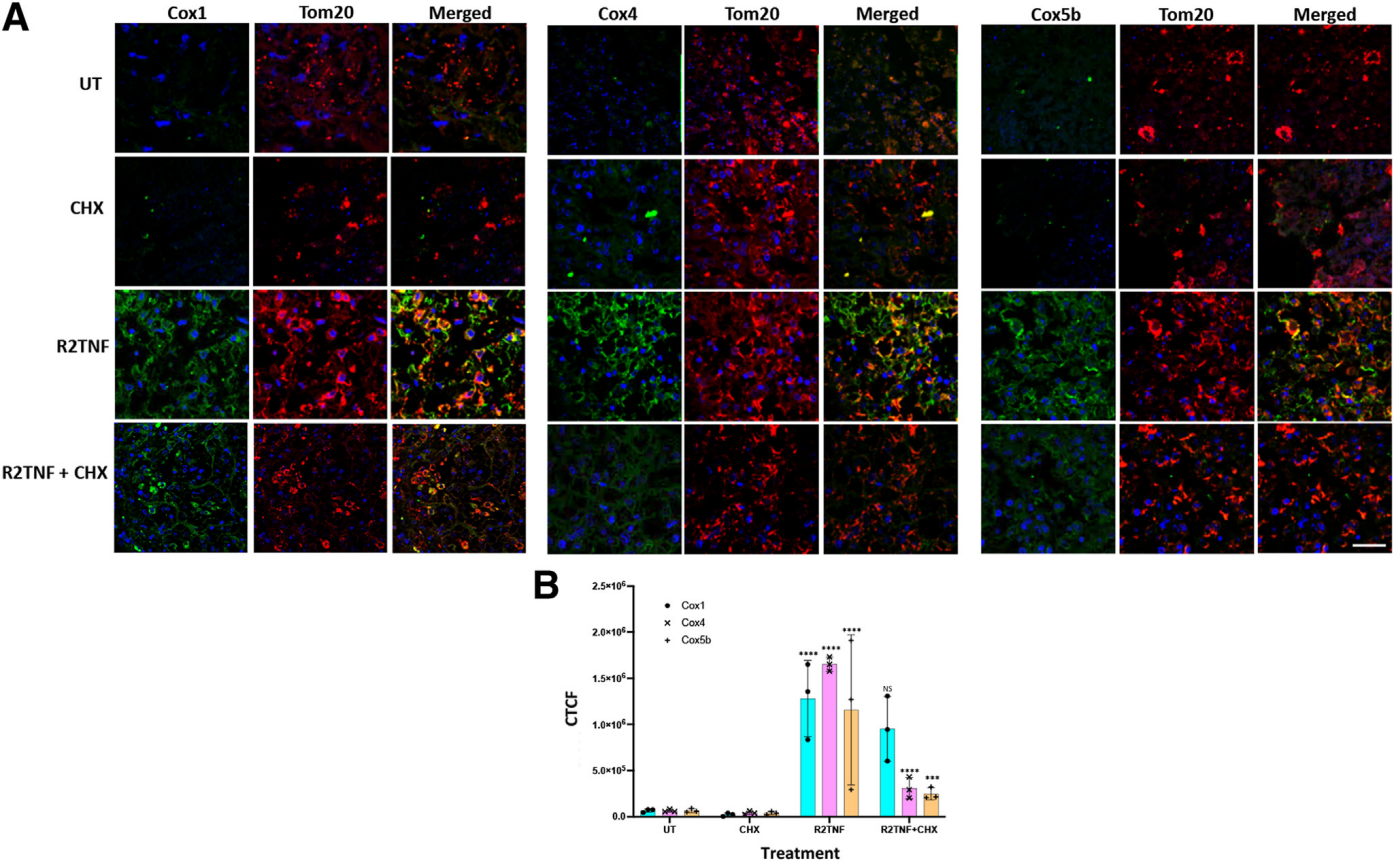
Cycloheximide (CHX) inhibits expression of nuclear-encoded cytochrome *c* oxidase (Cox) 4/5b but not the mitochondrially-encoded Cox1. **A:** Cultures pretreated with CHX (20 μg/mL) showed negligible levels of Cox1, Cox4, and Cox 5b comparable to untreated (UT) cultures. In comparison with UT, tumor necrosis factor receptor 2–specific mutein (R2TNF) alone induced increased levels of Cox1, Cox4, and Cox5b in tumor cells significantly reduced by CHX of Cox4 and Cox5b but no significant reduction of Cox1. Mitochondrial marker was translocase of outer mitochondrial membrane (Tom20). **B:** The positive signal in tumor cells is presented as corrected mean cell fluorescence (CTCF). Two-way analysis of variance was performed. Data are given as means ± SD (**B**). *n* = 3 per group with similar results (**B**). ∗∗∗*P* < 0.001 versus R2TNF (Cox5b); ∗∗∗∗*P* < 0.0001 versus UT or CHX or versus R2TNF (Cox4). Scale bar = 25 μm (**A**). Original magnification, ×40 (**A**). NS, not significant.

Cox1 expression may be modified by the cytoplasmic expression of nuclear-encoded Cox subunits.^28^ To further examine whether elevated expression of Cox1 is controlled by cytoplasmic translation, the cytoplasmic protein synthesis inhibitor cycloheximide was applied to RCC organ cultures.^29^ Immunostaining revealed that expression of Cox4/5b in mitochondria (confirmed by translocase of outer mitochondrial membrane co-localization) was profoundly inhibited by cycloheximide (R2TNF ± cycloheximide: Cox 4, approximately sevenfold reduction, *P* < 0.0001; Cox5b, approximately sixfold reduction, *P* < 0.001), whereas the expression of Cox1 was not significantly reduced (Figure 5A), as quantified (Figure 5B). Thus, the increase in Cox1 expression is likely to be controlled by either stable cytoplasmic factors or mitochondrially encoded mechanisms that are independent of cytoplasmic control.

### Elevated ^pSer65^-4EBP1 in ccRCC Tumor Cells Depends on mTORC1

**Figure 6:**
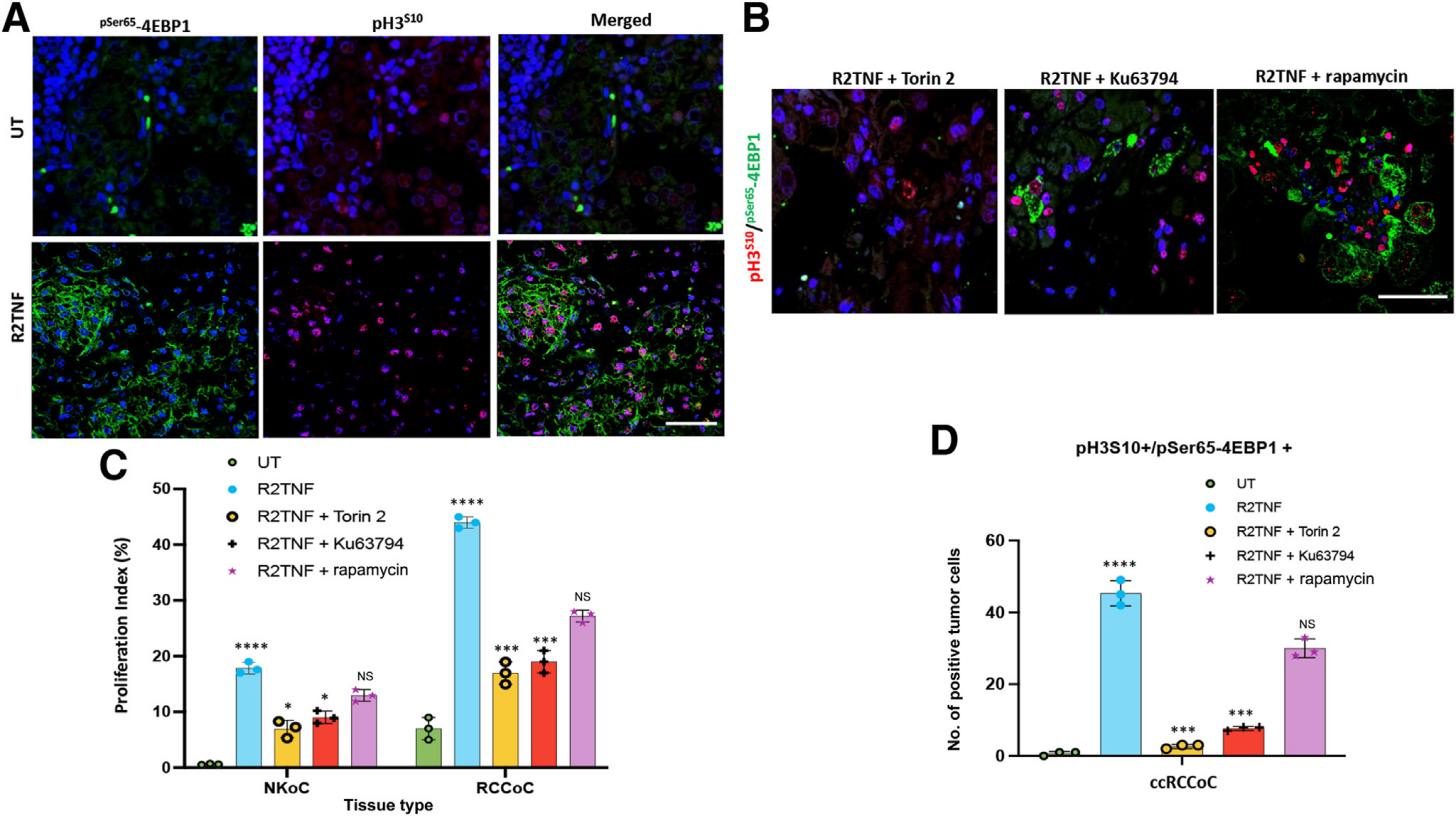
Tumor necrosis factor receptor 2–specific mutein (R2TNF)–induced cell proliferation and ^pSer65^-4EBP1 expression in tumor cells are reduced by mammalian target of rapamycin (mTOR) inhibitors. **A:** Representative confocal images of combined immunofluorescence for ^pSer65^-4EBP1 (green) and proliferative marker phosphorylated-histone S10 (pH3^S10^; red) in low-grade ccRCC organ culture either left untreated (UT) or treated with R2TNF for 3 hours at 37°C show a significant increase in proliferative tumor cells (pH3^S10+^) also positive for ^pSer65^-4EBP1 in R2TNF-treated cultures compared with UT. **B:** Similar cultures pretreated with mTOR inhibitors Torin 2, Ku63794, or rapamycin for 1 hour before R2TNF addition show a significant reduction in ^pSer65^-4EBP1^+^/pH3^S10+^ tumor cells, more pronounced in cultures pretreated with Torin 2 and Ku63794 than rapamycin. **C:** Quantification of tumor cell proliferation as a proliferative index (percentage positive of total cells) in UT or R2TNF-treated organ cultures of ccRCC (RCCoC) versus staining in normal tubular cells in organ cultures of nontumor tissue (NKoC) and in the absence or presence of the mTOR inhibitors. **D:** Quantification of double-stained ^pSer65^-4EBP1^+^/pH3^S10^ positive tumor cells in UT and R2TNF-treated cultures ± mTOR inhibitors. Two-way analysis of variance was performed. Data are given as means ± SD (**C** and **D**). *n* = 3 per group with similar results (**C** and **D**). ∗*P* < 0.05, ∗∗∗*P* < 0.001 versus R2TNF; ∗∗∗∗*P* < 0.0001 versus UT. Scale bars: 50 μm (**A**); 75 μm (**B**). Original magnifications: ×2.3 (**B**); ×40 (**C**). NS, not significant.

**Supplemental Figure S4:**
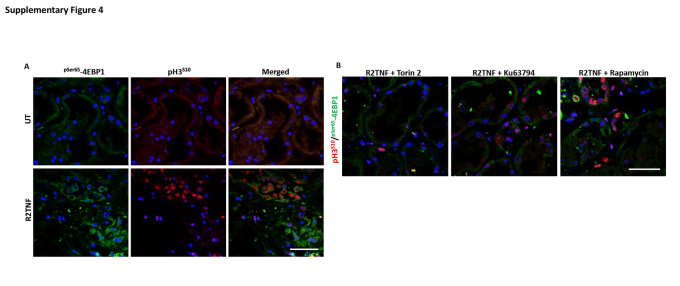
Tumor necrosis factor receptor 2–specific mutein (R2TNF)–induced cell proliferation and ^pSer65^-4EBP1 expression in organ culture of normal kidney reduced by mammalian target of rapamycin (mTOR) inhibitors. **A:** Representative confocal images of combined immunofluorescence for ^pSer65^-4EBP1 (green) and proliferative marker phosphorylated histone S10 (pH3^S10^; red) in normal kidney organ culture either left untreated (UT) or treated with R2TNF for 3 hours at 37°C show a significant increase in proliferative tubular cells (pH3^S10+^) also positive for ^pSer65^-4EBP1 in R2TNF-treated cultures compared with UT. **B:** Similar cultures pretreated with mTOR inhibitors Torin 2, Ku63794, or rapamycin for 1 hour before R2TNF addition show a significant reduction in ^pSer65^-4EBP1^+^/pH3^S10+^ tubular cells, more pronounced in cultures pretreated with Torin 2 and Ku63794 than rapamycin. *n* = 3 per group with similar results (**A** and **B**). Scale bars = 50 μm (**A** and **B**). Original magnification, ×40 (**A** and **B**).

**Supplemental Figure S5:**
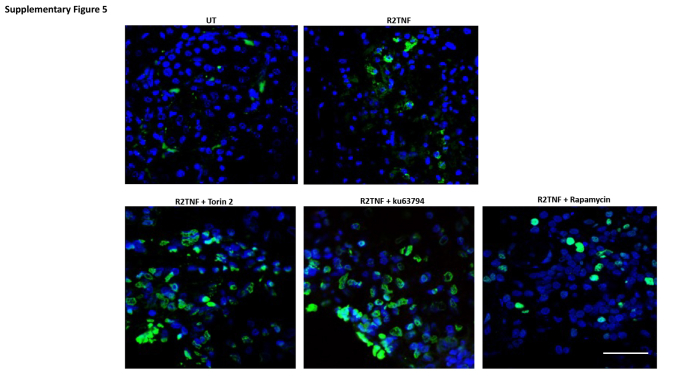
Representative confocal images of terminal deoxynucleotidyl transferase-mediated dUTP nick-end labeling staining in the absence and presence of the mammalian target of rapamycin inhibitors in organ cultures of low-grade ccRCC either left untreated (UT) or treated with tumor necrosis factor receptor 2–specific mutein (R2TNF). Images show significant cell death in cultures pretreated with Torin 2 and Ku63794 for 1 hour before R2TNF for 3 hours at 37°C compared with those pretreated with rapamycin. *n* = 3 per group with similar results. Scale bar = 50 μm. Original magnification, ×40.

Inhibition of the mTORC pathway inhibits TNFR2-induced cell cycle activation in cultured ccRCC^CD133+^ cancer stem-like cells.^6^^,^^7^ To examine whether the phosphorylation of 4EBP1 also correlates with TNFR2-induced cell cycle entry via mTORC signaling, organ cultures were costained for the proliferative marker pH3^S10^ and ^pSer65-^4EBP1. R2TNF treatment resulted in a more pronounced dual signal for ^pSer65-^4EBP1 and pH3^S10^ in tumor cells compared with untreated cultures (approximately 43.2% by R2TNF versus approximately 3.75% in UT cultures) (Figure 6A) as quantified (Figure 6C). R2TNF also induced a dual signal in normal tubular cells in organ cultures of NK, but the effect was significantly less than in organ cultures of ccRCC (approximately 17.6% by R2TNF versus approximately <1% in UT cultures (Supplemental Figure S4A), as quantified (Figure 6C). To test whether cell cycle entry depends on ^pSer65-^4EBP1, organ cultures were pretreated with the mTORC inhibitors Torin 2, Ku63794, or rapamycin. Torin 2 and Ku63794 significantly decreased the number of R2TNF-induced ^pSer65-^4EBP1^+^/pH3^S10+^ double-positive tumor cells in ccRCC (Figure 6B), as quantified (Figure 6D), with a potency of approximately 8.4-fold for Torin 2 and approximately 5.25-fold for Ku63794, in keeping with their pharmacologic potency,^30^ whereas rapamycin did not inhibit cell cycle significantly, as expected as it blocks the ribosomal protein S6 kinase 1 (S6K1) pathway but not the 4EBP1 arm of mTORC1 signaling.^30^ The inhibitors caused a similar but less effective inhibition of proliferation in organ cultures of NK (approximately 3.3-fold by Torin 2, approximately 2.2-fold by Ku63794, and nonsignificantly by rapamycin) but on a much lower signal intensity background (Supplemental Figure S4B), as quantified (Figure 6C). These results strengthen the notion that ^pSer65^-4EBP1 is necessary for cell cycle entry induced by R2TNF signaling in ccRCC. In keeping with previous observations,^7^ the decrease in cell proliferation in the presence of mTOR inhibitors was accompanied by an analogous increase in cell death measured using TUNEL staining (TUNEL^+^ cell increase of approximately fivefold induced by Torin, approximately threefold by Ku63794, and approximately twofold by rapamycin, although the latter was not significantly different from R2TNF-treated alone) (Supplemental Figure S5 and Table 3).

**Table 3:** Quantification of the Percentage of TUNEL^+^ Tumor Cells in Low-Grade ccRCCoC and of Tubular Cells in Corresponding NKoC Calculated as TUNEL^+^/Total Cells (×100) at ×40 Magnification

Treatment	NKoC	ccRCCoC
UT	0.9125 ± 0.1	3.41 ± 0.12
R2TNF	2.73 ± 0.3	8.90 ± 0.2∗
R2TNF + Torin 2	32.2 ± 0.5†	45.75 ± 0.7†
Torin 2	21.1 ± 0.3†	32.25 ± 0.32†
R2TNF + Ku63794	23.37 ± 0.2†	28.12 ± 0.8†
Ku63794	17.37 ± 0.6†	21.25 ± 0.6†
R2TNF + rapamycin	15.12 ± 1.2‡	18.37 ± 0.25‡
Rapamycin	9.75 ± 0.6‡	11.87 ± 0.6‡

Data are given as means ± SD percentages of three independent patient samples, NKoC, and ccRCCoC. Cells were either left UT or treated with R2TNF alone (1 μg/mL for 3 hours at 37°C) or pretreated with mammalian target of rapamycin inhibitors (all used at 50 μmol/L; 1 hour before R2TNF) or pretreated with inhibitors alone.

ccRCCoC, organ cultures of ccRCC; NKoC, organ cultures of nontumor kidney; R2TNF, TNFR2 mutein; TUNEL, terminal deoxynucleotidyl transferase-mediated dUTP nick-end labeling; UT, untreated.

∗ *P* < 0.05 versus UT.

† *P* < 0.0001.

‡ *P* < 0.001 versus R2TNF.

## Discussion

Previous studies^5^, ^7^ indicate that an important step in the TNFR2 signaling pathway that drives cell cycle entry in ccRCC involves crosstalk between phosphorylation of vascular endothelial cell growth factor receptor type 2, epithelial tyrosine kinase, PI3K, Akt, mTORC, and STAT3 on serine 727 (pStat3^Ser727^). This signaling is associated with the co-localization of pStat3^Ser727^ and TNFR2 within the mitochondria.^5^^,^^7^ In the present study, 4EBP1 phosphorylated serine 65 (^pSer65^-4EBP1) was identified as a significant mediator of mTORC signaling downstream of TNFR2. This conclusion is based on the following observations: i) In ccRCC tissue, ^pSer65^-4EBP1 expression relative to total 4EBP1 was significantly elevated in tumor cells compared with NK, with signal intensity increasing with malignant grade. ccRCC extracts also displayed enhanced 4EBP1 Thr37/46 phosphorylation. ii) In organ cultures, selective activation of TNFR2 by R2TNF, a specific TNFR2 ligand, increased ^pSer65-^4EBP1 expression to a greater extent in ccRCC compared with NK tissue. iii) mTORC1/2 inhibitors suppressed R2TNF-mediated induction of ^pSer65-^4EBP1 expression in correlation with their potency at inhibiting cell cycle entry, and increased tumor cell death.

The role of mTORC1 in the phosphorylation of 4EBP1 is well established and contributes to the increased level of protein synthesis mediated in response to mTORC1 activation. However, co-localization of ^pSer65-^4EBP1 with mitochondria (alongside TNFR2) and its association with the expression of the mitochondrially encoded COX subunit Cox1, as well as the nuclear-encoded Cox4/5 ab subunits, was unexpected. How these phenomena are linked mechanistically is unclear as mitochondrial ribosomes differ from cytoplasmic ribosomes and are not affected by cytosolic elongation factors. In keeping with this notion, cycloheximide inhibited 2RTNF-induced expression of nuclear-encoded Cox4 and Cox5b, whereas the increased expression of mitochondria-encoded Cox1 was not significantly reduced. Morita et al^17^ showed that mTORC-mediated 4EBP1 phosphorylation was essential for increasing ATP production by increasing the expression of nuclear-encoded mitochondrial genes in MCF7 cells, albeit not Cox4. Thus, it is possible that 4EBP1 can up-regulate mitochondrial function in ccRCC via increasing COX subunit expression. Whether the expression of other components of the mitochondrial respiratory machinery is also mediated by TNFR2 signaling via translational control remains to be seen. Taken together, results herein implicate a novel relationship between the TNFR2/^pSer65^-4EBP1/COX axis and mitochondrial function related to increased cell cycle activation in ccRCC.

Findings herein of increased TNFR2 expression in ccRCC are consistent with earlier reports,^5^^,^^7^ whereas the enhancement of tumor growth via TNFR2 is in line with findings by other groups^31^, ^32^, ^33^ and supports the notion that it is a promising target for cancer therapy.^34^^,^^35^ Although observation of TNFR2 localization with mitochondria in the present study is supported by previous reports,^36^, ^37^, ^38^ whether its effects on driving cell cycle entry are mediated via its mitochondrial localization requires further investigation. However, consistent with this hypothesis, previous studies have indicated a role for TNFR2 in mediating mitochondrial fusion, alongside increased mitochondrial oxidative phosphorylation activity^39^^,^^40^ and increased mitochondrial membrane potential, intracellular ATP levels, and oxygen consumption capacity.^41^

In many cancers, including ccRCC, the PI3K/Akt/mTORC1 pathway is aberrantly activated and contributes to oncogenesis, proliferation, invasion, and metastasis.^42^ mTORC1-dependent 4EBP1 phosphorylation alleviates the inhibition of eIF4E, releasing it to initiate cap-dependent mRNA translation.^43^ Augmented expression of phosphorylated 4EBP1 has been demonstrated in various human cancers, including RCC,^44^, ^45^, ^46^, ^47^, ^48^ suggesting it plays a pivotal role in tumorigenesis and is associated with adverse prognosis in several malignancies.^47^ Findings in the present study implicating 4EBP1 in mediating TNFR2-dependent tumor growth in ccRCC are that TNFR2 signaling increased the amount of phosphorylated 4EBP1 in ccRCC, in particular that of ^pSer65^-4EBP1, a final key phosphorylation site that enables the complete dissociation of 4EBP1 from eiF4E^26^^,^^49^^,^^50^ and that it is associated with TNFR2 localization in mitochondria. The role of ^pSer65-^4EBP1 in mediating cell cycle induction by TNFR2 in ccRCC is not completely resolved. However, observations in the present study of increased amount of mitochondrially encoded Cox1 in ccRCC organ cultures after TNFR2 stimulation, in addition to nuclear-encoded Cox4 and Cox5b, suggest that TNFR2-induced cell cycle entry in ccRCC is dependent on an increased energy demand that is met in part by mobilization of cytoplasmic and mitochondrial translational initiation complexes. That both nuclear and mitochondrially encoded COX protein subunits are elevated together is consistent with the observation that translation of mitochondrial Cox1 adapts to nuclear-encoded Cox4 availability,^28^ which may not necessitate mitochondrial localization of 4EBP1. The importance of COX subunits in driving mitochondrial energy production is well documented (reviewed by Timón-Gómez et al^51^). For example, depletion of Cox5b induces mitochondrial dysfunction by increasing reactive oxygen species production and decreasing mitochondrial membrane potential and intracellular ATP generation.^52^ In addition, Cox4 isoform switches enabled a cell to fine-tune COX function by altering ATP production, respiration, and mitochondrial reactive oxygen species production in response to tissue type, energetic status, and hypoxia.^53^^,^^54^ Singh et al^55^ have reported mitochondrial DNA-encoded Cox1 overexpression promotes anchorage-dependent and anchorage-independent proliferation potential in *in vitro* experiments, and somatic mutations within Cox1 and its perturbations from orchestrated expression influence carcinogenesis.

Organ culture experiments here demonstrate that the endothelial cells in ccRCC are more sensitive to TNFR2 signaling than the endothelial cells in the nontransformed parts of the kidney. Although the specific mechanisms responsible for this difference are unclear, an indirect mechanism that could support tumor growth would be by increasing angiogenesis. In endothelial cells, the TNF-α/TNFR2 pathway supports angiogenesis by activating the cytosolic tyrosine kinase epithelial tyrosine kinase that, in turn, phosphorylates and activates vascular endothelial cell growth factor receptor type 2 in the absence of vascular endothelial cell growth factor ligand, thereby promoting cell proliferation.^56^, ^57^, ^58^ Because phosphorylation of 4EBP1 plays a critical role in augmenting the cell's capacity for protein translation and angiogenesis,^59^ it is reasonable to suppose that the TNFR2/^pSer65^-4EBP1 axis plays a critical role in neovascularization of ccRCC in addition to its direct contribution to tumor cell growth.

Torin 2 and Ku63794 were found to significantly reduce TNFR2-triggered cell cycle activation with a noticeable reduction in expression of ^pSer65^-4EBP1 in tumor cells. Rapamycin's effects were not significant, in keeping with it being an inhibitor primarily of the S6K1 leg of the mTOR signaling pathway rather than the 4EBP1 leg.^30^^,^^60^^,^^61^ Interestingly, 4EBP1 phosphorylation has been implicated in rapamycin resistance in certain cancer cells^62^^,^^63^ and may explain the limited success of rapamycin as an anti-cancer drug.^64^ The residual activity observed in the presence of Torin 2 and Ku63794 may explain the resistance frequently seen with mTORC inhibitors used for the treatment of advanced RCC.^65^ One possibility is that TNFR2 mobilizes TORC2 as well as TORC1,^66^^,^^67^ and this pathway involves phosphorylation of Akt,^68^ which has previously been shown to be involved in driving TNFR2-mediated survival of CD133^+^ cancer stem-like cells together with vascular endothelial cell growth factor receptor type 2 and PI3K.^7^ Moreover, enhanced PI3K activity has been reported to promote protein synthesis and increase mTORC2 association with ribosomes, and 4EBP1 phosphorylation has been implicated in much of the activation of translation by PI3K/AKT, which plays an important part in mediating the effects of these pathways in tumors.^69^ In keeping with the possibility that 4EBP1 also mediates the AKT leg of the mTOR signaling pathway, a kinase activity specific to Ser65 and Thr70 on 4EBP1 was reported to be liberated from an mTOR immunoprecipitate, suggesting the presence of a different kinase.^70^ This observation suggests that an alternative TNFR2-4EBP1 kinase(s) is activated in response to mTORC inhibition, which may cause translational derepression from 4EBP1 in an mTORC-independent manner.

## Conclusions

**Figure 7:**
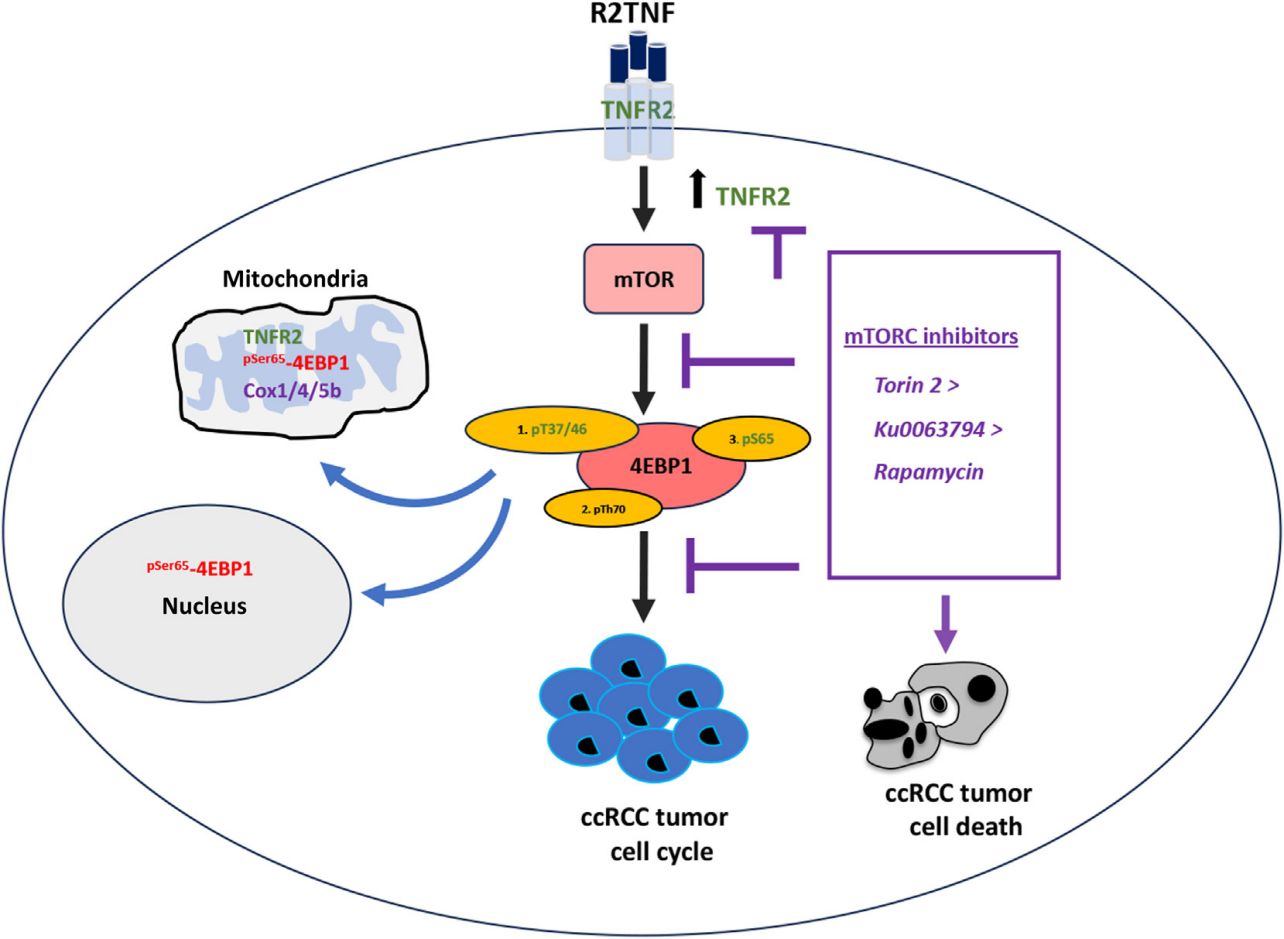
A model of tumor necrosis factor receptor (TNFR) 2–driven cell cycle entry in tumor cells in ccRCC. Selective ligation of TNFR2 by TNFR2-specific mutein (R2TNF) induces an increased expression of TNFR2 and phosphorylation of 4EBP1 at serine 65 (^pSer65^-4EBP1) and facilitates co-localization of the two proteins in cytoplasm and mitochondria alongside increased expression of nuclear- and mitochondrial-encoded cytochrome *c* oxidase (COX) subunits. ^pSer65^-4EBP1 is also detected in the nucleus in some tumor cells. Mammalian target of rapamycin (mTOR) inhibitors cause a significant reduction in R2TNF-mediated cell cycle activation and ^pSer65^-4EBP1 expression, accompanied by an increased cell death with the relative potency of Torin 2 > Ku63794 > rapamycin. mTORC, mTOR complex.

In summary, this study highlighted the importance of ^pSer65^-4EBP1 in TNFR2-driven cell cycle entry in tumor cells in ccRCC. It implicates mTOR-dependent and mTOR-independent pathways in this process, as schematized in Figure 7. The significant novel findings from the present study are the mitochondria-targeted ^pSer65^-4EBP1 and its association with TNFR2 in ccRCC, and the coordination between nuclear and mitochondrial expression of genes that enable increased mitochondrial performance.

## Disclosure Statement

None declared.
